# Genetic parameters and associated genomic regions for global immunocompetence and other health-related traits in pigs

**DOI:** 10.1038/s41598-020-75417-7

**Published:** 2020-10-28

**Authors:** Maria Ballester, Yuliaxis Ramayo-Caldas, Olga González-Rodríguez, Mariam Pascual, Josep Reixach, Marta Díaz, Fany Blanc, Sergi López-Serrano, Joan Tibau, Raquel Quintanilla

**Affiliations:** 1 Animal Breeding and Genetics Program, IRTA, Torre Marimon, 08140 Caldes de Montbui, Spain; 2 Department of Research and Development, Selección Batallé S.A., 17421 Riudarenes, Spain; 3 Université Paris‐Saclay, INRAE, AgroParisTech, GABI, 78350 Jouy‐en‐Josas, France; 4 IRTA, Centre de Recerca en Sanitat Animal (CReSA, IRTA-UAB), Campus de la Universitat Autònoma de Barcelona, 08193 Bellaterra, Spain; 5 Animal Breeding and Genetics Program, IRTA, Finca Camps i Armet, 17121 Monells, Spain

**Keywords:** Genetics, Animal breeding, Genetic association study, Genetic markers, Immunogenetics, Quantitative trait, Immunology, Immunogenetics, Innate immunity

## Abstract

The inclusion of health-related traits, or functionally associated genetic markers, in pig breeding programs could contribute to produce more robust and disease resistant animals. The aim of the present work was to study the genetic determinism and genomic regions associated to global immunocompetence and health in a Duroc pig population. For this purpose, a set of 30 health-related traits covering immune (mainly innate), haematological, and stress parameters were measured in 432 healthy Duroc piglets aged 8 weeks. Moderate to high heritabilities were obtained for most traits and significant genetic correlations among them were observed. A genome wide association study pointed out 31 significantly associated SNPs at whole-genome level, located in six chromosomal regions on pig chromosomes SSC4, SSC6, SSC17 and SSCX, for IgG, γδ T-cells, C-reactive protein, lymphocytes phagocytic capacity, total number of lymphocytes, mean corpuscular volume and mean corpuscular haemoglobin. A total of 16 promising functionally-related candidate genes, including *CRP*, *NFATC2*, *PRDX1*, *SLA*, *ST3GAL1*, and *VPS4A*, have been proposed to explain the variation of immune and haematological traits. Our results enhance the knowledge of the genetic control of traits related with immunity and support the possibility of applying effective selection programs to improve immunocompetence in pigs.

## Introduction

Over the last decades, the genetic selection in commercial pig breeds has greatly improved traits directly related with production performance^1^, while health-related traits have traditionally played a minor role in breeding programs. Nowadays, the emergence of antibiotic resistance and society demands for healthier livestock products and for more sustainable production systems^2^ represent new challenges for the pig production industry. Animal health is one of the most important contributors to productivity, profitability, and welfare, with multiple factors being involved in the maintenance of high health herd status such as co-infections of viral or bacterial pathogens, environmental stressors, and management practices. In the midst of strong investment for designing alternatives to antimicrobials in veterinary medicine^3^, the incorporation of health-related traits in pig breeding programs has become an emerging and challenging trend to produce more resilient, wellbeing and disease resistant pig populations.

Breeding approaches to improve animal robustness and disease resistance have been mainly focused on direct and indirect methods^4^. Direct methods usually rely on targeting the genetic resistance/susceptibility to specific diseases, requiring exposition to the infectious agents. This approach is expensive, time-consuming and information demanding. An indirect approach focused on the determination of the global immunocompetence of animals with no sign of infection has become a good alternative, but requires detailed knowledge of the different components of the immune system^4,5^. In this approach, immunity traits (ITs) may be considered as biologically relevant parameters to measure immunocompetence^4^. These traits may be classified into the two major components of the immune system, innate (or natural) immunity or acquired (adaptive) immunity, although there are also traits which are considered a bridge between both components^6^.

The innate immune system is the host’s first line of defence against infectious agents. In addition, haematological traits and stress parameters are also important indicators of the physiological and health status of farm animals^7–9^. During last years, several studies have reported medium to high heritabilities for several immune and haematological traits in pigs, suggesting an important genetic contribution to the phenotypic variability of these traits^4,10–14^. Since the pioneering work on quantitative trait loci (QTLs) mapping for general immune-capacity performed by^15^ in a wild boar × Swedish Yorkshire crossbred population, other groups have reported QTLs for traits related to immune-capacity in pigs^16–23^. More recently, with the development of high-density genotyping SNP chips, analyses applying genome wide association study (GWAS) have been performed to identify genetic markers associated with health-related traits. These studies have been however mainly addressed on haematological traits^24–34^. To date, genetic information in pigs on stress parameters focused primarily on acute adrenal activity^35–38^ with little or no genetic study on chronic stress parameters, such as cortisol measured in hair.

The present work aimed to study the genetic architecture of 30 health-related traits covering immune (mainly innate), haematological, and stress parameters associated to immunocompetence in a Duroc commercial line by estimating their genetic parameters and identifying associated genomic regions and candidate genes.

## Results

### Descriptive statistics and phenotypic correlations

In the present study we measured a set of 30 traits related with immune, haematological and stress parameters on a commercial Duroc pig line comprising 432 individuals. The data on descriptive statistics, as well as the abbreviated name of the analysed measured traits are shown in Table 1. Among the haematological traits, the erythrocyte-related traits presented the lowest phenotypic dispersion, with a coefficient of variation (CV) below 0.1, while the leukocyte and platelet-related traits presented CV ranging from 0.34 (leukocytes count) to 0.63 (eosinophils count). Regarding the ITs, most phagocytosis traits presented limited dispersion (CV ≤ 0.2), whereas the highest CVs were obtained for the acute-phase proteins CRP and HP (CV = 0.73 and 0.67, respectively). Finally, the stress parameters presented a moderate phenotypic variation with a CV = 0.44.

**Table 1:** Descriptive statistics of the analysed immunity, haematological and well-being traits.

Trait	Abrev	N	Mean	SD	CV
Haematocrit (%)	HCT	430	33.76	2.93	0.09
Haemoglobin (g/dL)	HB	430	10.64	0.94	0.09
Erythrocytes count n/µL	ERY	430	6,527,907	551,399	0.08
Mean corpuscular volume (fL)	MCV	430	51.83	3.47	0.07
Mean corpuscular haemoglobin (pg/cell)	MCH	430	16.34	1.15	0.07
Mean corpuscular haemoglobin concentration (g/dL)	MCHC	430	31.54	1.02	0.03
Platelets count n/µL	PLA	424	277,797	133,467	0.48
Leukocytes count n/µL	LEU	430	20,541.8	6919.3	0.34
Eosinophils count n/µL	EOS	430	314.59	196.95	0.63
Lymphocytes count n/µL	LYM	430	12,203.3	4472.4	0.37
Monocytes count n/µL	MON	430	532.93	279.14	0.52
Neutrophils count n/µL	NEU	430	7448.9	3309.2	0.44
IgA in saliva (mg/dl)	IgAsal	404	5.05	3.06	0.61
IgA in plasma (mg/ml)	IgA	432	0.65	0.31	0.48
IgG in plasma (mg/ml)	IgG	431	12.61	4.90	0.39
IgM in plasma (mg/ml)	IgM	432	2.26	0.82	0.36
C-reactive protein in serum (µg/ml)	CRP	428	173.01	126.01	0.73
Haptoglobin in serum (mg/ml)	HP	432	0.99	0.67	0.67
Nitric oxide in serum (µM)	NO	427	205.98	80.29	0.39
γδ T-lymphocyte subpopulation (%)	γδ T cells	396	7.97	5.03	0.63
Phagocytosis (% cells)	PHAGO_%	432	43.32	8.50	0.20
Granulocytes phagocytosis (%)	GRANU_PHAGO_%	432	91.74	3.81	0.04
Monocytes phagocytosis (%)	MON_PHAGO_%	432	49.90	9.74	0.20
Lymphocytes phagocytosis (%)	LYM_PHAGO_%	432	6.35	4.11	0.65
Phagocytosis FITC	PHAGO_FITC	432	4.69	0.33	0.07
Granulocytes phagocytosis FITC	GRANU_PHAGO_FITC	432	5.14	0.41	0.08
Monocytes phagocytosis FITC	MON_PHAGO_FITC	432	3.91	0.25	0.06
Lymphocytes phagocytosis FITC	LYM_PHAGO_FITC	432	3.16	0.13	0.04
Cortisol in hair (pg/mg)	CORT	431	166.91	72.62	0.44
NEU/LYM ratio	NLR	430	0.65	0.286	0.44

**Figure 1:**
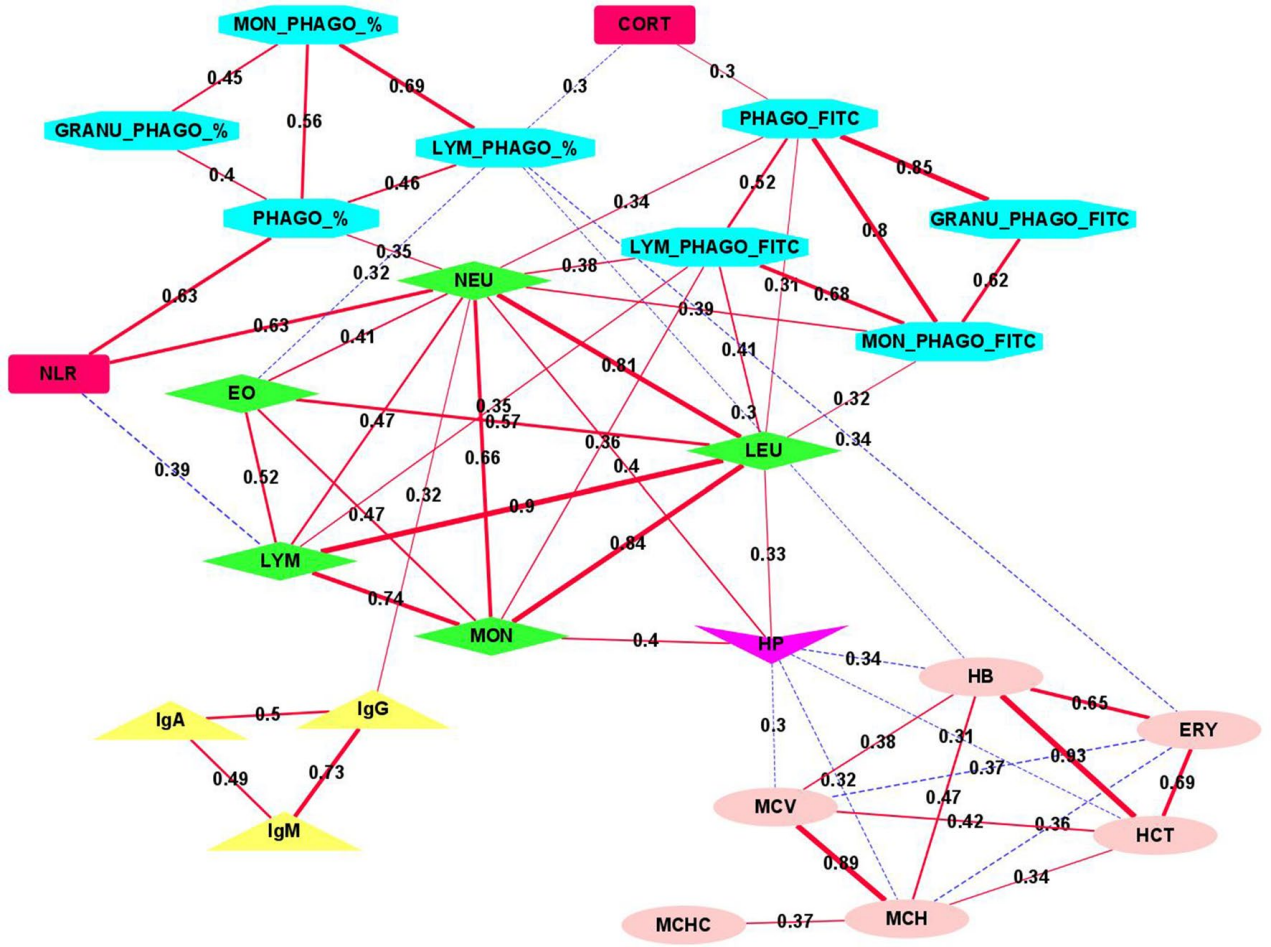
Network based on phenotypic correlation coefficients (|r| ≥ 0.3) among the immunity-related traits. Red lines indicate positive correlated traits while blue dashed lines indicate negative correlations. The numbers along the lines and the width of the lines indicate correlation coefficients. The shape and color of nodes indicate the different classes of the analysed traits.

The network based on phenotypic correlation coefficients (Fig. 1) identified five interconnected clusters of correlated traits. The central cluster grouped five hemogram leukocyte-related traits (LEU, NEU, EO, LYM and MON), with r_p_ ranging from 0.41 to 0.90. Another cluster connected with the previous one included traits related to phagocytic capacity (PHAGO_FITC, LYM_PHAGO_FITC, MON_PHAGO_FITC and GRANU_PHAGO_FITC) with r_p_ > 0.5 among them. The phagocytosis traits related to percentage of phagocytic cells (PHAGO_%, GRANU_PHAGO_%, LYM_PHAGO_%, MON_PHAGO_%) clustered also together. Finally, two more clusters were found, the plasma concentration of Ig (IgA, IgM and IgG), with a r_p_ = 0.73 between IgG and IgM, and the haematological erythrocyte-related traits (HB, ERY, MCV, MCH, HCT, MCHC), with negative and positive correlation coefficients. It is worth to highlight that HP generally correlated negatively with this last cluster. More details about the phenotypic correlation coefficients are provided in Supplementary Table S1.

### Genetic parameters of immunity-related traits: heritability and genetic correlations

The genetic determinism of immunocompetence was first explored by the heritability of these immunity and health-related phenotypes. Heritability (Table 2) ranged between 0.092 and 0.786. Most traits exhibited moderate to high heritability values, 22 out of 30 traits showing h^2^ values above 0.4. These heritabilities had relatively wide confidence intervals, that in some cases encompasses more than half heritability parameter space. Despite this, the h^2^ confidence intervals did not overlap zero but for the heritability of MON, MON_PHAGO_FITC and GRANU_PHAGO_%.

**Table 2:** Heritability values ($\widehat{{h}^{2}}$) for the immunity, haematological and well-being analysed traits, plus standard errors (SE) and confidence intervals at 95% (CI95) of the estimates.

Trait	$\widehat{{h}^{2}}$	SE	CI95
Haematocrit (%)	0.405	0.136	0.140–0.671
Haemoglobin (g/dL)	0.419	0.135	0.154–0.685
Erythrocytes count n/µL	0.669	0.133	0.408–0.930
Mean corpuscular volume (fL)	0.600	0.150	0.305–0.895
Mean corpuscular haemoglobin (pg)	0.470	0.135	0.205–0.736
Mean corpuscular haemoglobin concentration (g/dL)	0.767	0.150	0.473–1.061
Platelets count n/µL	0.651	0.153	0.351–0.951
Leukocytes count n/µL	0.281	0.128	0.030–0.533
Eosinophils count n/µL	0.594	0.143	0.314–0.875
Lymphocytes count n/µL	0.273	0.130	0.019–0.528
Monocytes count n/µL	0.092	0.080	− 0.065 to 0.248
Neutrophils count n/µL	0.640	0.149	0.348–0.932
IgA in saliva (mg/dl)	0.467	0.161	0.151–0.783
IgA in plasma (mg/ml)	0.671	0.132	0.412–0.930
IgG in plasma (mg/ml)	0.652	0.136	0.517–1.054
IgM in plasma (mg/ml)	0.786	0.137	0.386–0.919
C-reactive protein in serum (µg/ml)	0.245	0.114	0.119–0.685
Haptoglobin in serum (mg/ml)	0.402	0.144	0.021–0.469
Nitric oxide in serum (µM)	0.256	0.120	0.330–0.896
γδ T-lymphocytes subpopulation (%)	0.613	0.144	0.021–0.490
Phagocytosis (% cells)	0.425	0.146	0.139–0.710
Granulocytes phagocytosis (%)	0.185	0.103	− 0.016 to 0.386
Monocytes phagocytosis (%)	0.431	0.152	0.133–0.729
Lymphocytes phagocytosis (%)	0.495	0.149	0.202–0.788
Phagocytosis FITC	0.349	0.141	0.073–0.626
Granulocytes phagocytosis FITC	0.474	0.151	0.179–0.769
Monocytes phagocytosis FITC	0.118	0.106	− 0.089 to 0.325
Lymphocytes phagocytosis FITC	0.407	0.115	0.181–0.633
Cortisol in hair (pg/mg)	0.456	0.142	0.177–0.735
NEU/LYM ratio	0.731	0.155	0.427–1.035

Among analysed ITs, plasma concentrations of Ig showed the highest heritabilities (from 0.652 to 0.786), followed by the percentage of γδ T cells (h^2^ = 0.613). Focusing traits related to acute phase proteins, HP exhibited a relatively high heritability (h^2^ > 0.4), whereas more limited genetic contribution was estimated for CRP (h^2^ = 0.245) and also for NO (h^2^ = 0.256). For phenotypes related to phagocytosis, low to moderate heritabilities were obtained (from 0.118 to 0.495). Several haematological traits also exhibited high heritabilities, being the MCHC the most heritable among them (h^2^ = 0.767), followed by the quantity of ERY and PLA in blood and by the MCV (h^2^ ≥ 0.65 in both cases). Other erythrocyte-related phenotypes, such as total and mean corpuscular HB as well as HCT, also showed significant heritabilities above 0.4. Regarding white blood cells counts, the quantity of NEU and EOS in blood showed heritabilities above 0.55, whereas lowly values (h^2^ below 0.3) were obtained for the number of LYM and total LEU, and no significant additive genetic contribution to the number of MON could be assessed. Concerning stress parameters, a medium heritability (h^2^ = 0.456) was obtained for the CORT levels in hair (chronic stress indicator), whereas NLR showed a particularly high heritability (h^2^ = 0.731).

**Figure 2:**
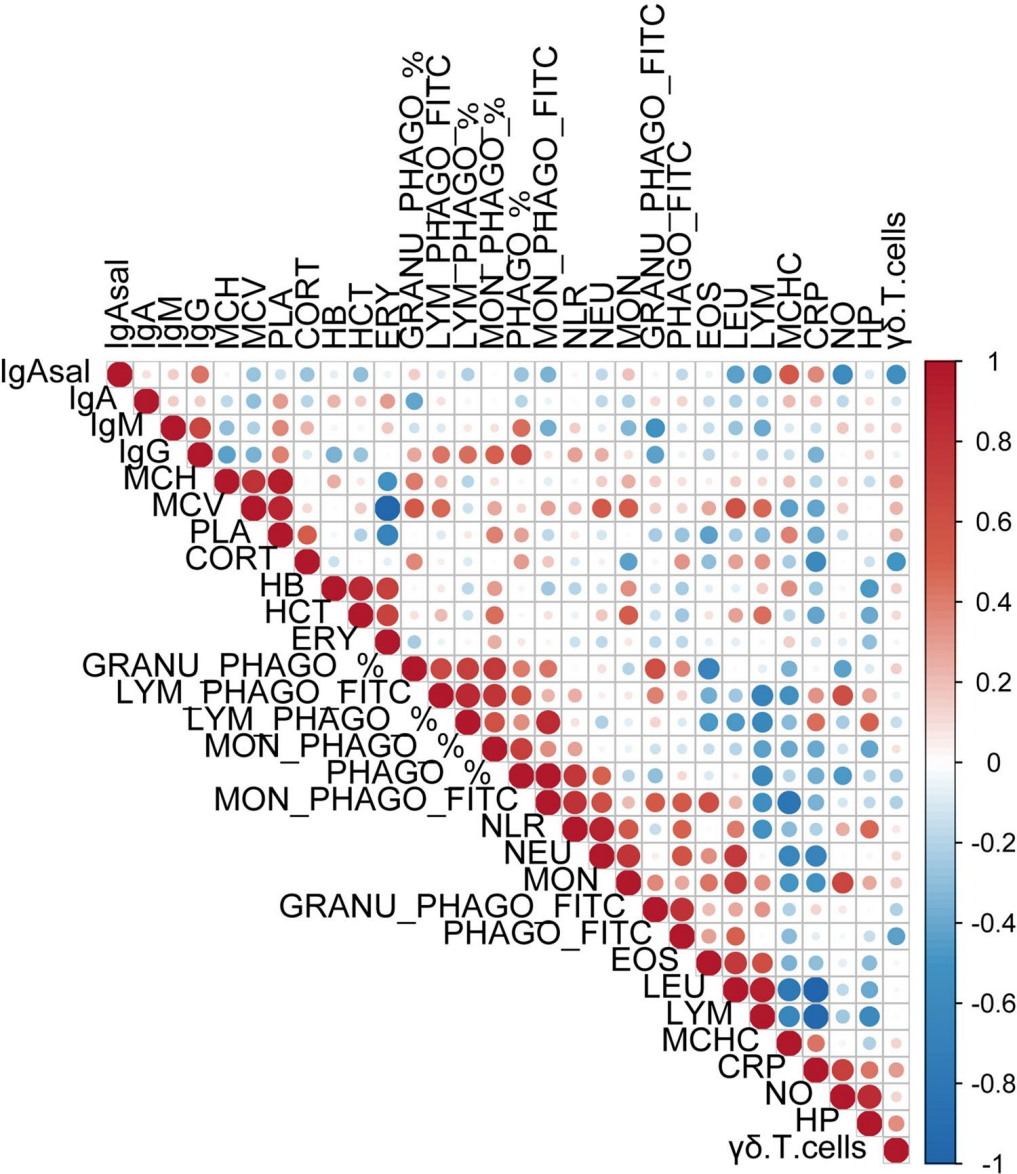
Heatmap of genetic correlations estimated by pairwise combination among immunity, haematological and stress related traits in pigs.

Genetic relationship among the immunity-related phenotypes was analysed through estimating the genetic correlations between each pairwise combination of traits; a heatmap showing the magnitude of the estimated correlations between the 30 traits is presented in Fig. 2. More details about these genetic correlation coefficients and their estimation standard errors are provided in Supplementary Table S2. It should be mentioned that the limited population size resulted in a limited precision of genetic correlations estimates, which generally showed high SE. The reliability of parameter estimation in the bivariate models was particularly compromised when they involved traits with heritabilities close to zero (i.e. MON, MON_PHAGO_FITC and GRANU_PHAGO_%), so these genetic correlations should be taken with caution.

The three plasma Ig (IgA, IgG and IgM) clustered together, showing positive genetic correlations among them but lower than phenotypic correlations (Fig. 1); only the genetic correlation between plasma IgM and IgG was relevant and significant (r_g_ = 0.662, SE = 0.118). In general, plasma Ig concentrations did not show important genetic correlations with other ITs but positive correlations between IgG and the percentage of phagocytic cells. Scarcely related with plasmatic Ig levels, the acute phase proteins concentrations in serum clustered together with NO and γδ T cells, with moderate to high positive genetic correlations among them but between γδ T cells and NO. CRP exhibited a very strong negative genetic associations with white blood cells counts, particularly LEU and LYM. Weaker but also negative genetic associations with red cells parameters (ERY, HB and HCT) were estimated for both CRP and HP.

A large cluster grouped together white cells counts and most phagocytosis capacity phenotypes quantified as mean fluorescence in FITC among the total phagocytic cells. The different leucocyte types (LYM, EOS, NEU, MON) showed a positive and high genetic correlation with total LEU count (r_g_ from 0.72 to 0.92), whereas genetic correlations between the different LEU types varied largely. Phagocytosis-related traits showed a complex and not always consistent (i.e. with large estimation error) picture of genetic associations. The FITC measurements of phagocytosis correlated positively between them and with leucocytes counts, with some exceptions involving lymphocytes that in turn showed a pattern of genetic associations relatively different from the rest of leukocyte types. Conversely, negative genetic associations between most leukocyte subsets and the proportion of phagocytic cells (PHAGO_%, GRANU_PHAGO_%, LYM_PHAGO_%, MON_PHAGO_%) were obtained. These percentages of phagocytic cells correlated positively between them (r_g_ between 0.35 and 0.75) but also with LYM_PHAGO_FITC and IgG.

As far as erythrocytes-related parameters is concerned, the HTC, HB and ERY constituted a cluster, with r_g_ between them ranging between 0.69 and 0.86. No evidences of relevant genetic associations between these traits and white blood cells were obtained but for HTC, that showed moderate positive genetic correlations with LYM, LEU and MON. Separately and opposed to the previous cluster, PLA, MCH and MCV also grouped together (r_g_ from 0.81 to 0.94); all three traits correlated negatively with ERY count.

Finally and regarding stress-related parameters, the CORT concentration in hair showed negative genetic correlations with CRP and γδ T cells (r_g_ =  − 0.59 and − 0.50, respectively) and positive correlation with PLA (r_g_ = 0.50); associations with the rest of haematological and ITs were generally weak and/or no significant. Regarding NLR, expectedly it was strongly correlated to NEU (r_g_ = 0.90), but also in a lesser extent to MON and LEU (r_g_ = 0.55 and 0.41, respectively), and negatively to LYM (r_g_ = − 0.52).

### Genomic regions and candidate genes associated with immunity traits, stress indicators and haematological parameters

**Figure 3:**
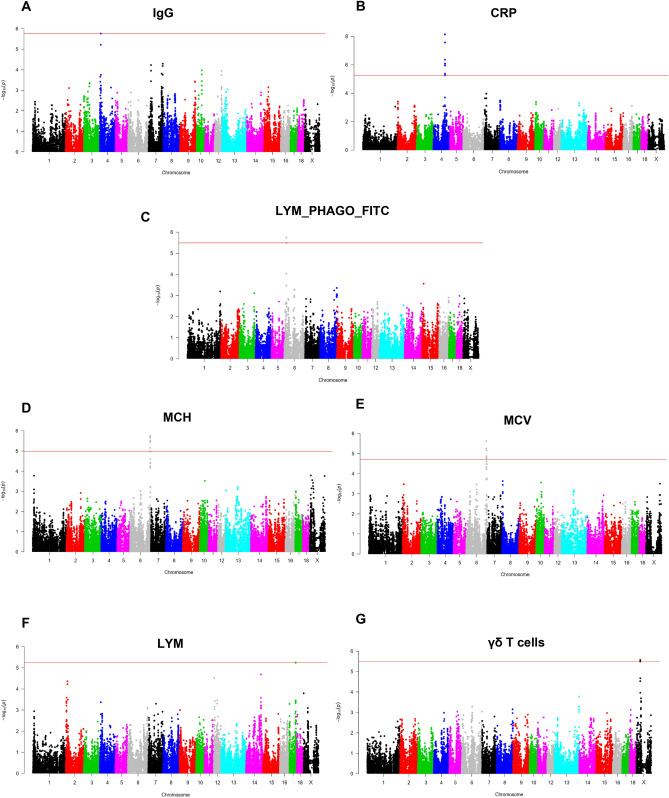
Manhattan plots representing the association analysis between the health-related traits and SNPs distributed along the pig genome. Red line indicates those SNPs that are below the genome-wide significance threshold (FDR ≤ 0.1).

To identify genomic regions associated with health-related traits, a GWAS was performed using the 30 phenotypic traits and the genotypes of 42,641 SNPs of the Porcine GGPSNP70 BeadChip (Illumina) in the 432 Duroc pigs. Significant associations at whole-genome level (FDR ≤ 0.1) were detected for IgG, γδ T cells, LYM_PHAGO_FITC, LYM, CRP, MCV and MCH. A total of 31 significant associated SNPs located in six chromosomal regions on pig chromosomes SSC4, SSC6, SSC17 and SSCX were identified (Table 3). In addition, a genomic region in SSC12 and SSC14 for the total number of LEU and in SSC13 for the total number of NEU passed the FDR threshold of < 0.2. In those regions, we identified nine associated SNPs (Table 3). The full list of associated SNPs, with their predicted consequences, is shown in Supplementary Table S3. In addition, graphical representation of the GWAS results for the traits are displayed in Manhattan plots in Supplementary Figure S1 (FDR ≤ 0.2) and Fig. 3 (FDR ≤ 0.1). It is worth to highlight that almost half of the QTLs identified (4 out of 9) were associated with lymphocytes-related traits.

**Table 3:** Description of the nine chromosomal regions associated with health-related traits and the annotated candidate genes.

Region	Chr	Start (Mbp)	End (Mbp)	N SNPs	Top SNPs	MAF	p-value	FDR	Trait	Candidate genes
1	4	8.39		1	rs319560097	0.44	1.71E−06	7.29E−02	IgG	*SLA*, *ST3GAL1*
2	4	90.54	91.26	10	rs81233340, rs81382318	0.15	7.17E−09	1.53E−04	CRP	*CRP*
3	6	17.11	17.18	2	rs338661853	0.50	1.78E−06	6.81E−02	LYM_PHAGO_FITC	*CDH1*, *COG8*, *VPS4A*
4	6	164.85	165.78	10	rs323656844	0.43	1.73E−06	2.18E−02	MCV, MCH*	*PRDX1*, *PIK3R3*
5	17	52.47	52.51	3	rs80924885, rs80899023, rs80803525	0.33	5.68E−06	8.07E−02	LYM	*NFATC2*
6	X	33.51	33.64	5	rs342772739	0.13	2.71E−06	2.71E−02	γδ T cells	ssc-mir-9786-1
7	12	3.25		1	rs323856019	0.17	3.26E−06	1.39E−01	LEU	*SOCS3*, *BIRC5*
8	13	69.03	71.96	7	rs81270251	0.48	9.01E−06	1.24E−01	NEU	*GATA2*, *PPARG*, *RAF1*, *SEC61A1*
9	14	123.89		1	rs343667976	0.45	9.07E−06	1.93E−01	LEU	–

* most significant trait

In SSC4, two regions at 8.3 Mb and 90.5–91.2 Mb associated with IgG plasma levels and CRP (Fig. 3A,B), respectively, were identified. In the proximal region of SSC4, an SNP (rs319560097) was associated with the IgG plasma levels. In this region, the candidate genes Src like adaptor (*SLA*) and ST3 beta-galactoside alpha-2,3-sialyltransferase 1 (*ST3GAL1*) related to B cell development, differentiation and function were identified. Ten SNPs in quite linkage disequilibrium (Dʹ = 0.203–0.999) were identified associated with CRP levels in a more distal region of SSC4. It is worth highlighting that two of these associated SNPs (rs81285109 and rs80958253) were located inside the *CRP* locus (Ensembl gene id: ENSSSCG00000006403), the main candidate gene identified in this region.

In SSC6, two regions at 17.11–17.18 Mb and 164.85–165.78 Mb were identified associated with three traits. In the proximal region of SSC6, two SNPs (rs338661853 and rs81285171) were associated with LYM_PHAGO_FITC (Fig. 3C). In this region, three candidate genes were annotated (*CDH1*, *COG8* and *VPS4A*), with the vacuolar protein sorting 4 homolog A (*VPS4A*) gene involved in the endosomal multivesicular bodies (MVB) pathway. In the second region, we identified ten SNPs in strong linkage disequilibrium (Dʹ > 0.9), except the rs81394075 that showed Dʹ > 0.48 with the rest of SNPs, associated with MCH and/or MCV traits (Fig. 3D,E). Remarkably, a strong candidate gene, the peroxiredoxin 1 (*PRDX1*), associated with haematocrit levels and haemoglobin concentration functions, was mapped in this pleitropic region. Another candidate gene also mapped in this region was the phosphoinositide-3-kinase regulatory subunit 3 (*PIK3R3*) which was identified as component of multiple canonical pathways of which erythropoietin signalling was among them.

Three SNPs (rs80924885, rs80899023, rs80803525) at 52.46–52.51 Mb of SSC17 were associated with LYM trait (Fig. 3F). In this region, we also identified a promising candidate gene, the nuclear factor of activated T cells 2 (*NFATC2*), directly related with the quantity of lymphocytes, quantity of T and B lymphocytes and size of thymus cortex functions. For the percentage of γδ T cells, five SNPs located at 33.51–33.64 Mb of SSCX were found to be significantly associated to this trait (Fig. 3G). In this region, we did not identify any candidate gene among the annotated protein coding genes. However, four lncRNA and ssc-mir-9786-1 were annotated in this region. Both types of RNAs have been directly implicated in the innate immune response^39,40^, although there is still a lack of information about the mechanism of action of lncRNAs. In contrast, miRNAs are better characterized and there are tools and databases that allow to perform an in-silico target prediction. In this way, ssc-mir-9786-1 was predicted to target a total of 528 genes by RNAhybrid. Noteworthy, among the biological functions represented in the list of targeted genes there was T cell differentiation (Supplementary Table S4).

Regarding LEU trait, two SNPs (rs323856019 and rs343667976) at 3.25 Mb and 123.89 Mb on SSC12 and SSC14, respectively, were associated with this trait. Two candidate genes (*SOCS3* and *BIRC5*) located on SSC12 and associated with quantity of leukocytes, lymphocytes, B and T lymphocytes, and peripheral blood leukocytes among other related diseases and functions were identified. On SSC14, no candidate genes were found among the annotated genes according to their functional information. Finally, seven SNPs with a Dʹ > 0.68 and located at 69.03–71.96 Mb on SSC13 were associated with NEU trait. In this region, several candidate genes (*GATA2*, *PPARG*, *RAF1* and *SEC61A1*) associated with functions such as quantity of leukocytes or neutropenia were identified.

### Comparison with other GWAS studies

A comparison was performed between the QTLs identified in our study and those previously published for health-related traits to identify overlapping chromosomal regions. We only identified two overlapping regions for LYM and MCH traits. Regarding LYM trait, the overlapping region (SSC17: 48.7–66.9 Mb) was identified in a F2 population from a Meishan/Pietrain family after 42 days post-infection with the protozoan pathogen *Sarcocystis miescheriana*^41^. The region for MCH at 166.3 Mb on SSC6 was less than 1 Mb away from the region identified in our analysis (164.8–165.8 Mb). This QTL was identified at chromosome-level in a GWAS analysis performed on animals of three breeds, Large-White, Landrace and Songliao Black^27^.

## Discussion

Robustness and resilience, together with well-being, are becoming a priority in livestock breeding. In our study, 30 traits including immunity, haematological and stress parameters have been measured in 432 healthy Duroc pigs to analyse individual’s immunocompetence. Most of these health-related traits presented medium to high heritability values, confirming a relevant genetic determinism in the phenotypic variation of the global immunocompetence in pigs, as had been suggested by several authors^10–14^. Overall, the heritabilities in our study are in close agreement with results previously reported in other pig populations^10–14^, very particularly those estimates showing high heritabilities for phagocytosis, γδ T cells, haematological erythrocyte-related traits, and IgA antibody levels, and the lower heritability for CRP. The LEU and LYM heritabilities in our study (low-to-medium) were also similar to those previously reported by^10–12,14^, but lower to the high heritabilities reported by^13^. In contrast, other haematological leukocyte-related traits (i.e. EO and NEU counts) together with HP and IgG antibody levels showed in our study higher heritabilities than those reported by^10–12^, but similar to the values of^13^. Finally, our heritability for total IgM antibody levels was higher than previously published^13^. Discrepancies with other studies were however not unexpected; they could be partially attributable to differences regarding age of animals, environmental factors or the lack of protocols standardization between laboratories, but it could also denote differences in the genetic determinism of immune capacity between different pig populations.

Besides former immunity and haematological-related traits, we also analysed the genetic determinism of two stress parameters, CORT and NLR, obtaining for them medium to high heritabilities. To the best of our knowledge, this is the first study reporting a heritability for the cortisol measured in hair, suggesting the existence of genetic determinism in the susceptibility of animals to chronic stress. In line with our results, some studies in humans determined low-to-medium heritabilities for acute plasma and saliva cortisol levels^42^, and post-ACTH cortisol levels in blood were reported to be highly heritable in pigs^43^.

Heritabilities previously stated would support the possibility of genetically improving the analysed immune and health-related traits in the studied Duroc pig population. Besides the possibility of applying a multi-trait selection for immune response (ability to respond immunologically) already reported in Yorkshire pigs by^44,45^, the alternative of applying selection on global immunocompetence of healthy animals is worthy to be explored^4,5^. The complex map of genetic interactions depicted by the estimated genetic correlations should be considered to design a global strategy to improve global immunocompetence for more robust and resilient pig populations. Estimates of genetic correlations between immunity and haematological traits reported here and in previous studies should be interpreted with caution, given the low estimabitity for such multi-trait models with limited sample sizes. We found however evidences of some relevant (and indisputably different from zero) genetic correlations. They allowed inferring a map of genetic associations among these traits that differed substantially from the phenotypic association map, and was in partial agreement with previous studies in other pig population (e.g.^13^), that generally found weak and mostly positive genetic correlations among ITs. In our study, we confirmed positive genetic correlations between several ITs, e.g. between IgG and IgM plasmatic concentrations or between leukocyte cells subsets, and of them with their phagocytosis capacity. But we also reported strong negative genetic correlations, for instance between leukocytes counts and CRP levels or between lymphocytes and percentage of phagocytic cells. Interestingly, the chronic stress indicator (CORT levels in hair) showed also genetic antagonism with relevant immunity parameters such as the percentage of γδ T-lymphocytes or CRP basal levels.

As signalled by^5^, major queries about the possibility and consequences of using genetic variation in immunocompetence in breeding programs should be addressed. According to our results, applying a selection program to increase the immunocompetence of the analysed Duroc population focusing for instance in lymphocyte related traits and/or immunoglobulins is feasible, but could be accompanied by correlated responses in other immunity parameters related to inflammation and stress that are worthy to be further explored. The question about putative correlated responses in (re)production performance should also be raised. Yorkshire pigs selected for high humoral and cellular immune responses had increased weight gains but were also prone to develop more severe arthritis after infection with *Mycoplasma hyorhinis*^44,45^. Further studies and functional validations are needed to determine the best combination of ITs and to assess the effects of selecting these ITs on global animal health and well-being, as well as on production performance. In this context and considering the time and cost-demanding phenotyping of ITs, the possibility of identifying genetic variants functionally related with immunity that could be implemented in the breeding schemes assumes paramount relevance.

GWAS analyses followed by gene annotation in the significantly associated genomic regions led to identify 16 promising candidate genes that may be implicated in the phenotypic variation of nine health-related traits. Remarkably, four out of nine of the traits with significant associated signals in the pig genome were related to lymphocytes, performing functions in the innate (percentage of γδ T cells in peripheral blood and lymphocytes phagocytic capacity) or the adaptive (total concentration of IgG in plasma and total number of lymphocytes) immune systems. In the opposite, our study did not allow identifying any SNP associated to CORT stress parameter. Genetic variants associated with plasma cortisol levels have been identified in pigs^35–38^, but there is a lack of GWAS studies with cortisol level measured in hair samples.

Among genes identified in the lymphocyte-signalled genomic regions, the *NFATC2* was mapped in the region associated with the total number of lymphocytes. This gene encodes a transcription factor that is expressed in peripheral blood lymphocytes, among others, and was firstly identified in T cells. NFATC2 plays a critical role in regulating the expression of cytokine genes in T cells during the immune response^46,47^ and is required for B cell development and function^46,48^. It is worth mentioning that knockout *NFATC2* mouse displayed enhanced immune response^49^ and hyperproliferation of primary B cells^48^, which suggest a negative regulatory function in the immune system.

Other two candidate genes, *SLA* and *ST3GAL1*, were located in the genomic region associated with the total concentration of IgG, the predominant serum isotype produced by B-lymphocytes. Remarkably, both genes have been implicated in the B cell differentiation process^50,51^. Specifically, expression of SLA is required to optimally regulate BCR levels and signal strength during B-cell development^50^, while *ST3GAL1* modulates the plant lectin peanut agglutinin (PNA) binding phenotype of activated B-cells, through O-glycan remodelling on CD45^51^.

As far as lymphocytes phagocytic capacity, three candidate genes were identified: cadherin 1 (*CDH1*), component of oligomeric golgi complex 8 (*COG8*) and *VPS4A*. Several studies have determined the phagocytic capacity of B-cells, mainly B1-cells but also follicular B-cells, playing an important role in innate immunity and the development of a strong humoral response^52–54^. VPS4A and COG8 have been involved in the generation of multivesicular bodies (MVBs) during phagosome maturation^55^, and retrograde intracellular membrane trafficking at the Golgi^56^, respectively. Furthermore, CDH1, a cellular receptor found on epithelial cells that can mediate entry of bacteria, is also expressed in other cells such as macrophages^57^.

Among the lymphocyte lineage there are cells such as the γδ T cells considered to be a bridge between innate and adaptive immunity^58^. Unlike in humans and mice, γδ T cells represent a prominent population in pigs’ peripheral blood^59^. In the genomic region associated with γδ T cells, we have identified a promising miRNA (ssc-mir-9786-1) which was predicted to target genes implicated in the T cell differentiation process. This miRNA was previously identified in porcine milk exosomes^60^ but there is still a lack of functional validation of the direct ssc-mir-9786-1-target mRNA interaction involving genes related with the immune system.

Also related to white cells-mediated immunity, we identified promising candidate genes annotated in the regions associated with the total number of leukocytes (SSC12 and SSC14) and neutrophils (SSC13). Remarkably, one of the candidate genes selected for the total number of leukocytes, baculoviral IAP repeat containing 5 (*BIRC5*), also known as *Survivin*, is essential for T cell maturation and proliferation^61^. This result is in accordance with the phenotypic and genetic correlations of r_p_ = 0.9 and r_g_ = 0.92 observed between the total number of leukocytes and lymphocytes. In fact, when we look in detail at the regions associated with the number of lymphocytes, the same signals previously observed for leukocytes are identified at chromosome level; therefore, these regions may be more specifically affecting lymphocytes. Among the candidate genes annotated in the region associated with NEU, it is worth to highlight the peroxisome proliferator activated receptor gamma (*PPARG*) gene. This gene encodes a nuclear hormone receptor with a wide variety of biological functions, including a critical role in modulating inflammatory processes of the innate immune system through regulation of neutrophil trafficking and apoptosis, among other functions^62^.

A particularly remarkable result arising in this study was the identification of *CRP* as candidate gene, annotated in the region associated with variation in its traduced protein levels. *CRP* is highly expressed during the acute-phase response, playing an important role in host defence through activating the complement system and cell-mediated pathways^63^. CRP is considered a blood biomarker of inflammation, although clinical studies in humans have determined that small elevation in baseline concentration of CRP is a powerful and specific predictor of cardiovascular event risk in healthy adults^64^. Remarkably, differences in CRP blood level have been associated with polymorphisms in the *CRP* gene, and some large-scale studies have provided evidence between the relationship of *CRP* polymorphisms, CRP blood levels and disease risk in humans (reviewed in^65^). In our study, we identified two associated SNPs in the intron 2 of the isoform ENSSSCT00000083957.1 and the 3′ UTR region (exon 2) of the isoform ENSSSCT00000007016.4. Further studies are warranted to determine the role of CRP polymorphisms in the variation of CRP serum levels in our Duroc population. Moreover, taking into account the higher resemblance of the immune responses of pigs with humans compared to mice^66^, the present results may contribute to the implementation of pigs as large animal models for cardiovascular diseases.

Finally, two interesting candidate genes (*PRDX1* and *PIK3R3*) were also identified in the region associated with both MCH and MCV. These red cell parameters are highly related, showing positive phenotypic and genetic correlations between them (r_p_ = 0.89, r_g_ = 0.81), which is concordant with the identification of this pleiotropic region. PRDX1 is a member of the peroxiredoxin family of antioxidant enzymes. Severe haemolytic anaemia characterized by marked decrease in haematocrit and haemoglobin in peripheral blood has been observed in mice lacking PRDX1^67^. Remarkably, MCV is among the 15 traits with the highest number of QTLs identified so far, with 546 associations (PigQTLdatabase, release 41, April 26, 2020). Nonetheless, we only identified a previously published QTL region associated with MCH^27^, which was proximal to the region for MCH/MCV identified in our study. This result agrees with previous studies in which few overlapping QTL regions for health-related traits have been identified so far, reinforcing the specificity of the genomic architecture of immunological parameters depending on the pig population (reviewed in^4^).

## Conclusions

This study focuses on the genetic basis of 30 phenotypes associated to health and well-being in a Duroc pig population. The medium-to-high heritability estimates confirmed the existence of genetic determinism in most traits related to global immunocompetence in pigs. Positive genetic correlations but also strong negative genetic correlations between several immunity traits were reported. We also identified nine chromosomal regions associated with the variation of nine immune traits, highlighting 16 promising candidate genes, including *CRP*, *NFATC2*, *PRDX1*, *SLA*, *ST3GAL1*, and *VPS4A*, functionally related to these traits. Overall, our results provide new insights into the genetic control of traits related with immunity and support the possibility of applying effective selection programs to improve immunocompetence in pigs.

## Methods

### Ethics statement

All experimental procedures with pigs were performed according to the Spanish Policy for Animal Protection RD1201/05, which meets the European Union Directive 86/609 about the protection of animals used in experimentation. The experimental protocol was approved by the Ethical Committee of the Institut de Recerca i Tecnologia Agroalimentàries (IRTA).

### Animal material

A total of 432 animals (217 males and 215 females) from a commercial Duroc pig line were used for this study. The pigs came from six batches (72 ± 1 animals per batch) and belonged to 134 litters (two to four piglets by litter, balancing gender when possible) obtained from 132 sows and 22 boars (all active boars in the commercial population). All animals were raised in the same farm and fed ad libitum with a commercial cereal-based diet. All animals were apparently healthy, without any sign of infection.

Samples of blood, saliva and hair were collected at 60 ± 8 d of age from all animals. Blood was collected via the external jugular vein into vacutainer tubes with or without anti-coagulants (Sangüesa S.A., Spain), according to the requirements for further immunity measurements. Saliva was collected with Salivette tubes (Sarstedt S.A.U., Germany) according to the protocols recommended by the manufacturer. Hair was collected with scissors from the dorsal area of the neck behind the ears and placed in numbered bags. All samples were transported with ice blocks to the laboratory for later processing.

### Phenotypic parameters

#### Haematological parameters

Hemograms were measured in the Laboratory Echevarne (Spain; Barcelona) from blood sampled in 4 ml EDTA tubes. The following haematological traits were included in the genetic analyses: haematocrit (HCT), haemoglobin (HB), mean corpuscular volume (MCV), mean corpuscular haemoglobin (MCH), mean corpuscular haemoglobin concentration (MCHC), total number of leukocytes (LEU), eosinophils (EO), lymphocytes (LYM), monocytes (MON), neutrophils (NEU), erythrocytes (ERY) and platelets (PLA).

#### Immunity parameters

Immunity parameters were measured from plasma or serum depending on the trait. Blood samples for serum were collected in 6 ml tubes with gel serum separator and centrifuged at 1600*g* for 10 min at RT. Plasma was collected from blood sampled in 6 ml heparinised tubes and centrifuged at 1300*g* for 10 min at 4 °C. Plasma and serum samples were collected, aliquoted, and stored a − 80 °C until use.

##### Immunoglobulins

Total concentrations of immunoglobulins IgA, IgG and IgM in plasma, and IgA in saliva, were measured by ELISA with commercial kits (Bethyl laboratories Inc., Bionova, Spain), following the manufacturer’s instructions. Plasma samples were diluted 1:10,000, 1:50,000 and 1:500,000 to detect IgA, IgG and IgM, respectively, while saliva samples were diluted 1:100 to detect IgA. Samples, in duplicate, were quantified by interpolating their absorbance from the standard curves constructed with known amounts of each pig immunoglobulin class and corrected for sample dilution. Absorbance was read at 450 nm using an ELISA plate reader (Bio-Rad) and analysed using the Microplate manager 5.2.1 software (Bio-Rad).

##### Acute-phase proteins

C-reactive protein (CRP) levels were measured in serum samples diluted 1:3000 by ELISA kit (Abcam Plc., Spain) following manufacturer’s instructions. Haptoglobin (HP) concentration was measured in undiluted serum samples by colorimetric assay (Tridelta Development Limited, Ireland) following manufacturer’s instructions. All samples were quantified in duplicate using standard curves constructed by plotting absorbance against CRP or HP concentration, respectively. Absorbance was read at 450 nm for CRP and 630 nm for HP using an ELISA plate reader (Bio-Rad) and analysed using the Microplate manager 5.2.1 software.

##### Gamma-delta T cells (γδ T cells)

Peripheral blood mononuclear cells (PBMCs) were separated from heparinised peripheral blood by density-gradient centrifugation with Histopaque-1077 (Sigma, Spain) at 450 g for 30 min. The cells were resuspended in RPMI 1640 medium supplemented with 5% foetal bovine serum (FBS) (Sigma, Spain), 1% Penicillin–Streptomycin (10,000 U/mL–10 mg/mL) and 1% l-Glutamine (200 mM) (Cultek, Spain). For PBMCs staining, the monoclonal antibody APC Rat Anti-Pig γδ T Lymphocytes (MAC320 clone, BD Pharmigen, Spain) and the APC Rat IgG2a κ isotype control (R35-95 clone, BD Pharmigen, Spain) were used. Briefly, 10^6^ PBMCs were stained with the primary-conjugated antibodies (1:100) in 1×PBS-1% FBS for 20 min at 4 °C. After two washes with 1×PBS-1% FBS at 4 °C, cells were resuspended in 1×PBS-1% FBS and analysed by flow cytometry using the MACSQuant Analyzer 10 Flow cytometer (Miltenyi Biotec GmbH, Bergisch Gladbach, Germany) and the MACSQuantify sofware v2.6 (Miltenyi Biotec GmbH, Bergisch Gladbach, Germany). For automated flow cytometry analysis, files were imported in R environment (v3.6.1)^68^ with the read.flowSet function implemented in flowCore package (v1.50.0)^69^. Fluorescence was transformed using arcsinhTransform function. Doublets were removed using gate_singlet function (flowStats package v3.42.0^70^), margin events using boundaryFilter function (flowCore package v1.50.0^69^). Gatings were then performed using gate_mindensity2 function (openCyto package v1.22.2^71^) on FSC channel to remove FSC low events corresponding to debris, SSC high events corresponding to residual granulocytes and to gate γδ T cells as APC positive. Parameters were adjusted for each day of lab analyses on a representative sample pooling all the data into one using getGlobalFrame function.

##### Phagocytosis assay

Phagocytosis assay was carried out in heparinized whole blood samples incubated with fluorescein (FITC)-labelled opsonized *E. coli* bacteria by using the Phagotest kit (BD Pharmigen, Spain) and according to the protocol recommended by the manufacturer. Samples were analysed by flow cytometry using the MACSQuant Analyzer 10 Flow cytometer (Miltenyi Biotec GmbH, Bergisch Gladbach, Germany) and the MACSQuantify sofware v2.6 (Miltenyi Biotec GmbH, Bergisch Gladbach, Germany). With this assay we analyzed the percentage of cells having performed phagocytosis as well as their mean fluorescence intensity (number of ingested bacteria). Phagocytosis assay analyses were performed in R (v3.6.0)^68^. Doublets were removed using gate_singlet function (flowStats package v3.42.0^70^), margin events using boundaryFilter function (flowCore package v1.50.0^69^). Gatings were then performed using either gate_mindensity2 or gate_flowClust_2d functions (openCyto package v1.22.0^71^) on propidium iodure (PI) channel to gate blood cells as PIhi and cells having performed phagocytosis as FITC+ . Granulocytes, monocytes, and lymphocytes were gated based on their FSC SSC properties. Parameters were adjusted for each day of lab analyses on a representative sample pooling all the data into one using getGlobalFrame function. The following phagocytosis traits were quantified: percentage of total phagocytic cells (PHAGO_%), percentage of phagocytic cells among granulocytes (GRANU_PHAGO_%), monocytes (MON_PHAGO_%), and lymphocytes (LYM_PHAGO_%), mean fluorescence in FITC among the total phagocytic cells (PHAGO_FITC), and mean fluorescence in FITC among the granulocytes (GRANU_PHAGO_FITC), monocytes (MON_PHAGO_FITC) and lymphocytes (LYM_PHAGO_FITC) that phagocyte.

##### Nitric oxide

Total concentrations of Nitric Oxide (NO) were measured by colorimetric assay (Thermo Fisher Scientific, Spain) following manufacturer’s instructions. Serum samples were ultrafiltered through a 10,000 molecular weight cut-off (MWCO) and diluted 1:10. Samples were quantified by reference to standard curves constructed with known amounts of Nitrate Standard solution. Absorbance was read at 540 nm using a microplate reader (LUMistar Omega, BMG Labtech) and analysed using the Omega MARS software (BMG Labtech).

#### Stress indicators

##### Cortisol

One hundred and fifty mg of hair were weighted from each sample and placed into a 50-ml conical tube. After three washes with 3 ml of isopropanol, all samples were left to dry in an extractor hood during 12 h. Dried hair samples were cut into 2–3 mm pieces using scissors, and 50 mg were transferred into 2 ml eppendorf. One ml of methanol was added to each sample and incubated 18 h at 37 °C under moderate shaking (100 rpm). After incubation, extracted samples were centrifuged at 7000*g* for 2 min and 700 µl of supernatant was transferred to a new 1.5 ml tube. The supernatant was then placed into a speed vac for 2 h to evaporate the methanol. The dried extracts were stored at − 20 °C until analysis. Total concentrations of cortisol (CORT) were measured by ELISA kit (Cusabio Technology LLC., Bionova, Spain) with dried samples reconstituted with 210 µl of sample diluent. Samples were quantified by reference to standard curves constructed with known concentrations of pig cortisol dilutions of the Standard. Absorbance was read at 450 nm using an ELISA plate reader (Bio-Rad) and analysed using the Microplate manager 5.2.1 software (Bio-Rad).

##### Neutrophil to lymphocyte ratio

The neutrophil to lymphocyte ratio (NLR) was calculated as a ratio of NEU and LYM.

### Exploratory and phenotypic analyses

Descriptive statistics of the formerly described immunity, haematological and well-being traits in our studied Duroc population are shown in Table 1. Exploratory analyses of these phenotypes were carried out for investigating both the raw data distribution and the best fitting model for subsequent analyses. Systematic non-genetic putative effects (sex, batch and day of lab analyses within batch) on each trait were tested by using a linear model (lm) in R. Normal probability plots and Shapiro–Wilk test were performed to investigate the goodness-of-fit of the residuals with the normal distribution. For most phenotypes but the percentage of phagocytic cells, data in raw form and its residuals were quite skewed to the right; in those cases, log-transformation of data corrected these departures from normality. A filtered dataset of log-transformed data (most phenotypes) and raw-data (% of phagocytosis) was employed to perform further analyses. Subsequently, pairwise phenotypic correlations (r_p_) among all analysed phenotypes were computed after adjusting for significant environmental factors, and a correlation network was built up with Cytoscape^72^, considering those Pearson’s correlation coefficients with absolute value ≥ 0.3.

### Estimation of genetic parameters

The heritability $({h}^{2})$, i.e. the proportion of phenotypic variance attributable to additive genetic effects, was estimated for the 30 immune, haematological and stress traits showed in Table 1. Variance components and the corresponding h^2^ were estimated from an univariate animal model as follows:

$${\varvec{Y}}={\varvec{X}}{\varvec{\beta}}+{\varvec{Z}}{\varvec{u}}+{\varvec{e}}$$

where ${\varvec{Y}}$ is the vector of phenotypic observations of all individuals for the health-related trait (either log-transformed or raw data, depending upon the trait); ${\varvec{\beta}}$ is the vector of systematic (fixed) effects on the trait, including effect of sex (2 levels) plus batch effects (6 levels) for most traits but for phagocytosis-related traits, for which the data of laboratory analysis (12 levels, two by batch) was considered instead; ${\varvec{X}}$ is the corresponding incidence matrix; ${\varvec{u}}$ is the vector of animal’s genetic additive (random) effects on the trait, and ${\varvec{Z}}$ the corresponding incidence matrix; and ${\varvec{e}}$ is the vector of random residual terms. The assumed distribution of additive genetic effects was **u**∼*N*(0,**A** ${\sigma }_{u}^{2}$), where **A** is the numerator relationship matrix computed on the basis of pedigree (1388 individuals, five generations) and ${\sigma }_{u}^{2}$ is the additive genetic variance; random errors were distributed as **e** ∼ *N*(0,**I** ${\sigma }_{e}^{2}$). Estimation of the model variance components and the corresponding heritability $({h}^{2}= {\sigma }_{u}^{2}/\left({\sigma }_{u}^{2}+{\sigma }_{e}^{2}\right))$ for each trait was performed by REML using the *aireml* program included in the BGF90 package^73^; the standard errors (SE) of the heritability estimates were computed by repeated sampling from their asymptotic normal distribution following^74^, thus obtaining the corresponding confidence intervals at 95% (CI95).

Subsequently, pairwise genetic correlations (for each two traits combination) were estimated in a two-traits animal model described as follows in matrix notation:

$$\left \lceil\begin{array}{c}{{\varvec{Y}}}_{t1}\\ {{\varvec{Y}}}_{t2}\end{array} \right \rceil= \left \lceil\begin{array}{cc}{{\varvec{X}}}_{t1}& 0\\ 0& {{\varvec{X}}}_{t2}\end{array}\right \rceil\left[\begin{array}{c}{{\varvec{\beta}}}_{t1}\\ {{\varvec{\beta}}}_{t2}\end{array}\right]+ \left \lceil\begin{array}{cc}{{\varvec{Z}}}_{t1}& 0\\ 0& {{\varvec{Z}}}_{t2}\end{array} \right \rceil\left[\begin{array}{c}{{\varvec{u}}}_{t1}\\ {{\varvec{u}}}_{t2}\end{array}\right]+\left[\begin{array}{c}{{\varvec{e}}}_{t1}\\ {{\varvec{e}}}_{t2}\end{array}\right]$$

where ${{\varvec{Y}}}_{t1}$ and ${{\varvec{Y}}}_{t2}$ are the vectors of phenotypic observations for trait 1 and trait 2, respectively; ${{\varvec{\beta}}}_{t1}$ and ${{\varvec{\beta}}}_{t2}$ are the vectors of systematic (fixed) effects on each trait previously described, and ${{\varvec{X}}}_{t1}$ and ${{\varvec{X}}}_{t2}$ the correspondent incidence matrices; ${{\varvec{u}}}_{t1}$ and ${{\varvec{u}}}_{t2}$ are the vectors of animal genetic additive effects on trait 1 or trait 2 (random effects), and ${{\varvec{Z}}}_{t1}$ and ${{\varvec{Z}}}_{t2}$ the corresponding incidence matrices; finally ${{\varvec{e}}}_{t1}$ and ${{\varvec{e}}}_{t2}$ are the vectors of residual errors for each trait, and **0** a matrix of zeros, assumed independent. The (co)variance matrix of random genetic effects was defined as:

$$Var\left[\begin{array}{c}{{\varvec{u}}}_{t1}\\ {{\varvec{u}}}_{t2}\end{array}\right]= \left \lceil\begin{array}{cc}{\varvec{A}}{\sigma }_{u1}^{2}& {\varvec{A}}{\sigma }_{u1,u2}\\ {\varvec{A}}{\sigma }_{u1,u2}& {\varvec{A}}{\sigma }_{u1}^{2}\end{array} \right \rceil$$

where $${\sigma }_{u1}^{2}$$ and $${\sigma }_{u2}^{2}$$ are the additive genetic variance of traits 1 and 2, respectively, $${\sigma }_{u1,u2}$$ is the genetic covariance between the traits, and **A** is the numerator relationship matrix as defined above. Estimation of the (co)variance components of each pairwise analysis was also performed by REML using the *aireml* program included in the BGF90 package^73^. Genetic correlation between traits were obtained as  $${{\text{r}}_{\text{g}}} = \left( {{\raise0.7ex\hbox{${{\sigma _{u1,u2}}}$} \!\mathord{\left/ {\vphantom {{{\sigma _{u1,u2}}} {{\sigma _{u1}}{\sigma _{u2}}}}}\right.\kern-\nulldelimiterspace} \!\lower0.7ex\hbox{${{\sigma _{u1}}{\sigma _{u2}}}$}}} \right)$$, and the SE of the genetic correlation estimates were also computed following^74^.

### DNA extraction and SNP genotyping

Genomic DNA was extracted from blood samples using the NucleoSpin Blood (Macherey–Nagel). DNA concentration and purity were measured in a Nanodrop ND-1000 spectrophotometer.

The 432 animals were genotyped for 68,516 single nucleotide polymorphisms (SNPs) with the GGP Porcine HD Array (Illumina, San Diego, CA) using the Infinium HD Assay Ultra protocol (Illumina). Plink software^75^ was used to remove SNPs with a minor allele frequency (MAF) less than 5%, SNPs with more than 10% missing genotype data, and SNPs that did not map to the porcine reference genome (Sscrofa11.1 assembly). After quality control a subset of 42,641 SNPs were retained for subsequent analysis.

### Genome-wide association studies (GWAS)

GWAS was carried out between the 42,641 filtered SNPs and the 30 health-related traits described in Table 1. For this purpose, the genome-wide complex trait analysis (GCTA) software^76^ was employed using the following model for each trait across all SNPs:

$$y_{ijk} = sex_{j} + b_{k} + u_{i} + s_{li} a_{l} + e_{ijk}$$

where *y*_*ijk*_ corresponds to the phenotypic trait (either log-transformed or raw data) of the *i*th individual of sex *j* in the *k*th batch; *sex*_*j*_ corresponds to the *j*th sex effect (2 levels); *b*_*k*_ corresponds to the *k*th batch effect (6 levels) for most traits but for phagocytosis related traits, for which the data of laboratory analysis (12 levels, two by batch) was considered instead; *u*_*i*_ is the infinitesimal genetic effect of individual *i*, with **u**∼*N*(0,**G**σ^2^_*u*_), where **G** is the genomic relationship matrix (GRM) calculated using the filtered autosomal SNPs based on the methodology of^76^ and σ^2^_u_ is the additive genetic variance; *s*_*li*_ is the genotype (coded as 0,1,2) for the *l*th SNP, and *a*_*l*_ is the allele substitution effect of the SNP on the trait under study; and *e*_*ijk*_ is the residual term. The false discovery rate (FDR) method of multiple testing described by Benjamini and Hochberg^77^ was used to measure the statistical significance for association studies at genome-wide level with the *p.adjust* function of R. The significant association threshold was set at FDR ≤ 0.1. Manhattan plots based on the significance of the associations across the whole genome were generated using the R package qqman^78^.

Comparative QTL analysis between our results and previous published data was performed by retrieving all pig QTL and association data on SSC11.1 for health traits from the pigQTL database^79^.

### Gene annotation and SNP functional prediction

Biomart software^80^ was used to retrieve gene annotations from the Ensembl Genes 98 Database using the Scrofa11.1 reference assembly, considering 1 Mb downstream/upstream of around the candidate chromosomal regions. Furthermore, functional predictions of the significantly associated SNPs were performed with Variant Effect Predictor software^81^.

For functional categorisation of the annotated genes, data were analyzed through the use of IPA^82^ (QIAGEN Inc., https://www.qiagenbioinformatics.com/products/ingenuitypathway-analysis) to obtain gene ontologies (GO), biological functions, gene networks and canonical pathways in which the genes annotated in the associated regions were involved. Orthologous human gene names were retrieved from the Ensembl Genes 98 Database for functional categorisation when a pig gene name was not assigned to the gene stable id. Furthermore, information from Mouse Genome Database^83^ and Genecards^84^ was used to identify gene functions affecting the analysed phenotypes.

### miRNA target prediction and functional annotation

Porcine mRNA 3′UTR sequences from the whole pig genome (assembly 11.1) were downloaded from Ensembl Gene 100 database^85^, and Seqkit tool^86^ was used to search 3′ UTR seed matches with the 7mer seed miRNA sequence. Subsequently, the obtained list of 3′ UTR was used to predict miRNA target using the RNA hybrid software^87^ with the following criteria: energy threshold of no more than − 25 kcal/mol and perfect match of 2–8 nt in the seed region. Enriched GO terms and pathways of the predicted miRNA target genes was performed with the ClueGO v2.5.7 plug-in of Cytoscape v3.8.0 software^88^.

## Supplementary information

Supplementary Information.

Supplementary Table S1.

Supplementary Table S2.

Supplementary Table S3.

Supplementary Table S4.

## Data Availability

The results from all data generated or analysed during this study are included in this published article (and its Supplementary Information files). However, the datasets used and/or analysed during the current study are available from the corresponding author on reasonable request.
