# Supplementary material for: Genetic parameters and associated genomic regions for global immunocompetence and other health-related traits in pigs
Source: Sci Rep. 2020 Oct 28;10:18462. doi: 10.1038/s41598-020-75417-7 (PMC7595139; doi:10.1038/s41598-020-75417-7)
Supplement: Supplementary file 1 — Supplementary Information. [file 41598_2020_75417_MOESM1_ESM.docx]

**SUPPLEMENTARY MATERIAL**

**Genetic parameters and associated genomic regions for global immunocompetence and other health-related traits in pigs**

Maria Ballester^1*^, Yuliaxis Ramayo-Caldas^1^, Olga González-Rodríguez^1^, Mariam Pascual^1^, Josep Reixach^2^, Marta Díaz^2^, Fany Blanc^3^, Sergi López-Serrano^4^, Joan Tibau^5^, Raquel Quintanilla^1*^

^1^Animal Breeding and Genetics Program, IRTA, Torre Marimon, E08140 Caldes de Montbui, Spain.

^2^ Department of Research and Development, Selección Batallé S.A., E17421 Riudarenes, Spain.

^3^Université Paris‐Saclay, INRAE, AgroParisTech, GABI, 78350 Jouy‐en‐Josas, France.

^4^IRTA, Centre de Recerca en Sanitat Animal (CReSA, IRTA-UAB), Campus de la Universitat Autònoma de Barcelona, 08193 Bellaterra, Spain.

^5^ Animal Breeding and Genetics Program, IRTA, Finca Camps i Armet, E17121 Monells, Spain.

*E-mail: [maria.ballester@irta.cat](mailto:maria.ballester@irta.cat); [raquel.quintanilla@irta.cat](mailto:raquel.quintanilla@irta.cat)

**Figure S1**. Manhattan plot representing the association analysis between the health-related traits and SNPs distributed along the pig genome. Blue line indicates those SNPs that are below the genome-wide significance threshold (FDR≤0.2).


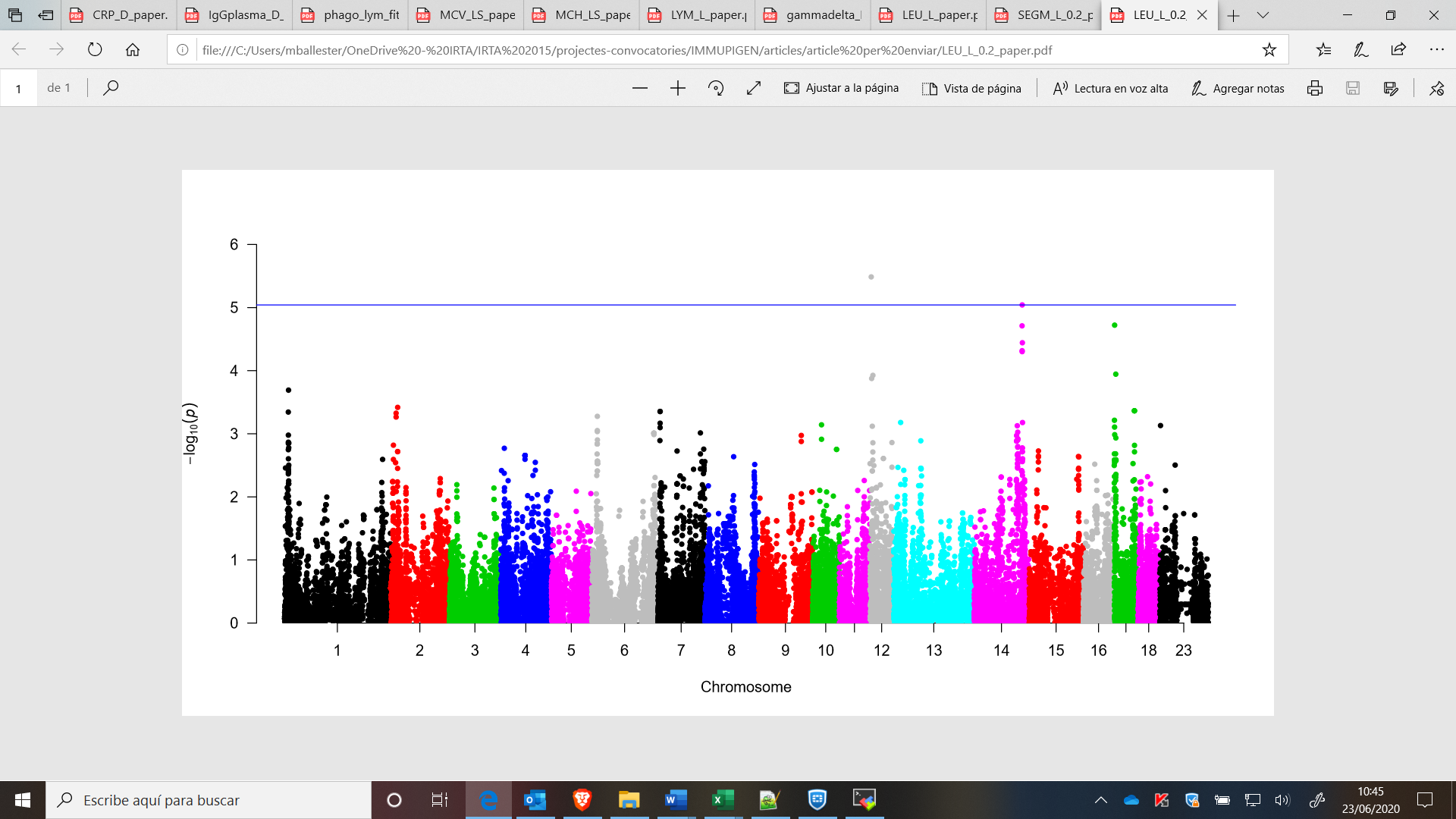


**LEU**


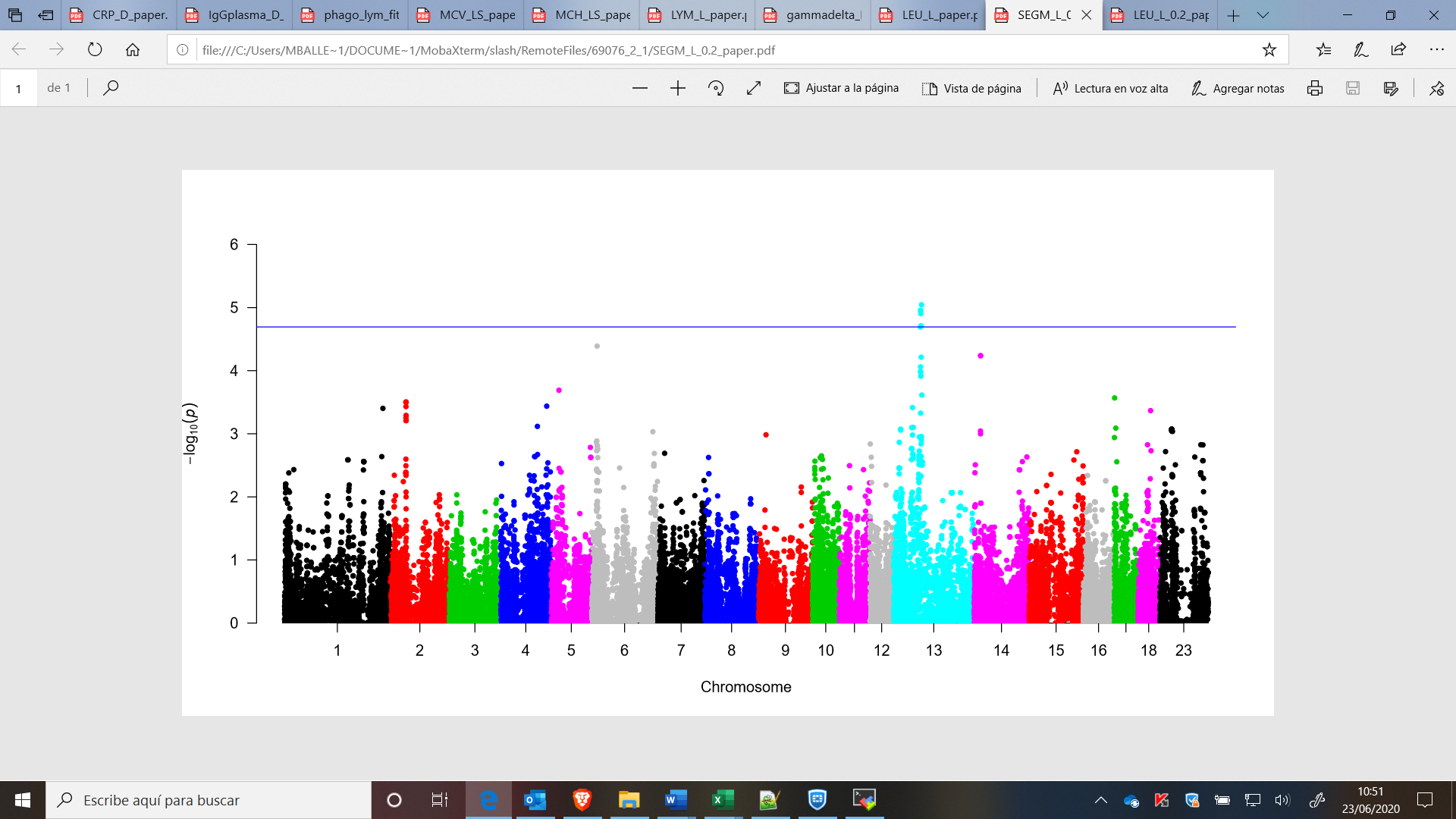


**NEU**

**Table S1.** Phenotypic correlation coefficients among the health-related traits.

**Table S2.** Genetic correlation coefficients and their estimation standard errors among the health-related traits.

**Table S3.** Description of the 40 associated SNPs with their predicted consequences.

**Table S4.** List of biological functions of predicted ssc-mir-9786-1 targeted genes.
